# What Cues Do Ungulates Use to Assess Predation Risk in Dense Temperate Forests?

**DOI:** 10.1371/journal.pone.0084607

**Published:** 2014-01-03

**Authors:** Dries P. J. Kuijper, Mart Verwijmeren, Marcin Churski, Adam Zbyryt, Krzysztof Schmidt, Bogumiła Jędrzejewska, Chris Smit

**Affiliations:** 1 Mammal Research Institute, Polish Academy of Sciences, Białowieża, Poland; 2 Department of Environmental Sciences, Copernicus Institute, Utrecht University, Utrecht, The Netherlands; 3 The Polish Society for Bird Protection, Białystok, Poland; 4 Community and Conservation Ecology Group, Centre for Evolutionary and Ecological Studies, University of Groningen, Groningen, The Netherlands; Institut Pluridisciplinaire Hubert Curien, France

## Abstract

Anti-predator responses by ungulates can be based on habitat features or on the near-imminent threat of predators. In dense forest, cues that ungulates use to assess predation risk likely differ from half-open landscapes, as scent relative to sight is predicted to be more important. We studied, in the Białowieża Primeval Forest (Poland), whether perceived predation risk in red deer (*Cervus elaphus*) and wild boar (*Sus scrofa*) is related to habitat visibility or olfactory cues of a predator. We used camera traps in two different set-ups to record undisturbed ungulate behavior and fresh wolf (*Canis lupus*) scats as olfactory cue. Habitat visibility at fixed locations in deciduous old growth forest affected neither vigilance levels nor visitation rate and cumulative visitation time of both ungulate species. However, red deer showed a more than two-fold increase of vigilance level from 22% of the time present on control plots to 46% on experimental plots containing one wolf scat. Higher vigilance came at the expense of time spent foraging, which decreased from 32% to 12% while exposed to the wolf scat. These behavioral changes were most pronounced during the first week of the experiment but continuous monitoring of the plots suggested that they might last for several weeks. Wild boar did not show behavioral responses indicating higher perceived predation risk. Visitation rate and cumulative visitation time were not affected by the presence of a wolf scat in both ungulate species. The current study showed that perceived predation risk in red deer and wild boar is not related to habitat visibility in a dense forest ecosystem. However, olfactory cues of wolves affected foraging behavior of their preferred prey species red deer. We showed that odor of wolves in an ecologically equivalent dose is sufficient to create fine-scale risk factors for red deer.

## Introduction

Prey species commonly react to increased levels of predation risk by showing anti-predator behavior, such as increasing vigilance levels or avoiding risky habitats [1]. These anti-predator responses often come at the cost of foraging [2] resulting in sub-optimal resource use [3]. For this reason it has been suggested that changes in herbivore prey behavior in response to carnivore presence may be of similar [4] or even greater importance for modifying herbivore-plant interactions than density-mediated effects of carnivores on their prey [3,5,6]. 

Much of our knowledge regarding effects of carnivores on wild ungulate behavior and subsequent effects on lower trophic levels originates from North American study systems. For example, studies from the Yellowstone National Park (YNP) illustrated that reintroduction of gray wolves (*Canis lupus*) in 1995 has resulted in the re-establishment of a ‘landscape of fear’, in which prey constantly adjust their behavior in response to spatially and temporally varying predation risk [6]. Three main anti-predator behavioral responses in elk (*Cervus elaphus canadensis*) were observed following wolf reintroduction: alteration of vigilance level and foraging time [6], changed group size and group sex composition [7-9] and changed habitat preference [10-12]. These observed changes in ungulate behavior have been suggested to result in decreased browsing intensity by elk on tree species in habitats with high predation risk, such as riparian valleys [13-15]. However, other authors have argued that these indirect (behaviorally-mediated) effects play only a minor role relative to direct (density-mediated) effects of wolves on their prey in explaining patterns of tree regeneration [16-18]. There is consensus that behaviorally-mediated effects of predators on ungulates do occur. However, the scale at which these effects operate is less clear. While several studies have illustrated effects of predator presence on large-scale distribution of ungulates, undisputed evidence that ungulates react to fine-scale risk factors affecting foraging behavior or patch selection on a small scale is lacking [18]. 

The underlying mechanisms of these observed behavioral changes in ungulate communities are still poorly understood. Especially, it remains unclear on which basis prey assesses or perceives predation risk induced by large carnivores. This can be either based on habitat features or cues that directly indicate predator presence [19] or a combination of both. Landscape characteristics clearly influence predator-prey interaction and the expression of risk effects [20]. Prey can use the long-term risk level of a habitat to make decisions on anti-predator behavior, such as increasing vigilance or avoiding risky habitats. However, it is not always easy to generalize how habitat or landscape characteristics drive perceived predation risk. Ungulates in both North American and African ecosystems have been shown to avoid and to be more vigilant in habitats with low visibility and high incidence of escape impediments [21-26]. In ecosystems with typical *ambush* predators, such as lions (*Panthera leo*) or Eurasian lynx (*Lynx lynx*), these are also the habitats where most carnivore kills occur [27-29]. In contrast, prey killed by wolves (a *chase* predator) often occur in open, grassy areas where visibility is high and escape impediments are lacking [20,30], which is in line with the observed large-scale movement of elk in YNP away from open, grassy areas after wolf introduction [11,12,30]. Stronger anti-predator response in open habitats versus closed habitats was also found in roe deer (*Capreolus capreolus*) exposed to Iberian wolf predation [31]. These differences in reaction of ungulates to habitat visibility are at least partly related to the carnivore hunting mode, where ambush predators would increase predation risk in areas with low visibility, and chase predators would create the opposite effect. Studies addressing how the effects of different types of predators combine in areas where they co-occur are rare [32]. Moreover, the question is whether habitat visibility plays a similar role in different ecosystems. The African and American ecosystems where relationships with visibility have been demonstrated are characterised by half-open landscapes, with large contrasts between low-visibility and high visibility habitats. Whether ungulates use habitat visibility as a cue when they solely occur in more dense, closed habitats, such as forest ecosystems, is the question.

Besides habitat features, prey can assess predation risk on the basis of cues indicating predators presence [19]. For example, elk have been shown to change vigilance levels, movement and grouping patterns on a short timescale when wolves were in the direct vicinity [8,19,30]. Yet, it remains untested which predator cues (e.g. sightings, sounds or odor of predator) and what intensity is sufficient to trigger a reaction in wild ungulates towards their natural predators. Recent experimental studies showed that enclosed Père David's deer (*Elaphurus davidianus*) reacted to both visual and acoustic cues of their potential predators [33]. Besides, a larger number of studies showed the importance of olfactory cues. The addition of caracal (*Felis caracal*) droppings was found to reduce foraging behavior of free-ranging domestic goats [34]. Furthermore, wolf scat suppressed feeding in food-choice experiments in domestic sheep [35] and the vigilance level of domestic cows increased when exposed to wolf scat [36]. Browsing damage by deer has also been shown to decrease when trees were sprayed with predator urine or scat extracts [37,38]. These studies illustrated that ungulates clearly react to predator olfactory cues, but evidence comes from either domestic ungulates or has been tested in unnatural settings (e.g. predator odor added to feeding bowls) or with unnatural high concentrations of predator odor. The question thus remains whether predator odor in an ecologically meaningful concentration and under field conditions provides a possible mechanism for changes in wild ungulate behavior. Especially in dense forest ecosystems with low visibility, olfactory cues are a likely factor associated with perceived predation risk of ungulates.

 The present study aimed at testing which cues ungulates use to assess predation risk in a lowland temperate deciduous forest ecosystem (Białowieża Primeval Forest, BPF, eastern Poland). Our study system contrasts with well-studied carnivore-ungulate systems in North-America and Africa as it is composed mainly of dense forest, lacking open or half-open habitats. We therefore predict that habitat visibility is an unlikely factor related to perceived predation risk by ungulates as a relatively small range in visibility is predicted to occur. Moreover, the two large carnivores which occur in BPF, wolves (as a coursing predator) and lynx (ambush predator), are expected to have opposing effects in relation to predation risk in low and high visibility habitats. Olfactory cues indicating predator presence are more likely used by ungulates to assess predation risk in these low visibility habitats. We studied the effects of habitat visibility and predator olfactory cues on two of the most numerous ungulate species in our system: red deer (*Cervus elaphus*) and wild boar (*Sus scrofa*). We predicted that behavioral changes associated with higher perceived predation risk (higher vigilance, see 6,8,24) should be more pronounced in red deer than in wild boar, as the latter does not play a major role in the diet of both predators [39,40] and predation plays a smaller role as a mortality factor in wild boar compared to red deer [41,42]. Moreover, we predicted that olfactory cues of a predator in a concentration equivalent to natural conditions should be sufficient to evoke a behavioral response of red deer.

## Methods

### Ethics Statement

The Director of the Białowieża National Park granted permission for this study in the strictly protected area. Permissions to carry out this study in the managed part of the forest were granted by the administration of the Polish State Forestry (Białowieża, Hajnówka and Browsk Districts). Since all data collected on vertebrates (ungulates) did not involve endangered or protected species and were based on non-invasive sampling (video cameras), no permission from ethical commissions was required. Our habitat visibility measurements were based on a non-destructive method and did not require further permission. The owners of the captive wolves had all required permissions to keep these animals and collection of scat occurred without disturbance of the wolves. 

### Study area

Our study was performed in the Białowieża Primeval Forest (BPF), a large continuous temperate mixed lowland forest of 1450 km^2^, which is located on the border of Poland and Belarus. The Polish part (52°45’ N, 23°50’ E) of the BPF (600 km^2^) consists of the Białowieża National Park (BNP) of 105 km^2^ and an adjacent managed forest. At present, BNP includes a 47.5 km^2^ area of strictly protected old-growth forest in which no human intervention (including forestry activities and hunting) has been allowed since 1921. Before 1921, human impact on tree stand structure and composition was small or minimal [43,44]. The climate is continental with a mean annual temperature of 6.8°C. The coldest month is January with on average -4.7 °C and the warmest month is July with 17.8 °C. Mean annual precipitation is 641 mm and mean annual snow cover lasts for 92 days. 

 The BPF consists of rich multispecies tree stands with five main forest types occurring along gradients of soil richness and water availability: deciduous forest (dominant tree species: *Quercus robur, Tilia cordata* and *Carpinus betulus*), mixed deciduous forest (*Picea abies, Quercus robur, Tilia cordata* and *Carpinus betulus*), black alder bog forest and streamside alder-ash forest (*Alnus glutinosa* and *Fraxinus excelsior*), mixed coniferous forest (*Pinus sylvestris*, *Picea abies* and *Quercus robur*) and coniferous forest (*Pinus sylvestris* and *Picea abies*) [45,46]. The managed part of the forest differs from the strictly protected stands inside the BNP in tree species composition, with more coniferous forest and a younger age class-distribution of the tree stands [47]. The majority of the area is covered by forest interspersed by small river valleys, marsh lands and forest gaps. Inside the BNP only 0.8 % of the area is lacking tree cover [48]. 

 A unique feature of the BPF is that it is one of the few areas in Europe, where the native assemblage of ungulates still occurs (five species) together with their natural predators. The most abundant species in the BPF both in numbers and crude biomass is red deer [43]. During the most recent survey based on drive counts in January 2011 (T. Borowik, unpubl. data) in the managed part of the BPF, a winter density of 4.7 individuals km^-2^ was recorded. The second-most numerous ungulate was wild boar with 3.2 individuals km^-2^, followed by roe deer with 0.8 individuals km^-2^. The larger species European bison (*Bison bonasus*) and moose (*Alces alces*) occur in the lowest densities, with 0.8 and 0.06 individuals km^-2^, respectively. Ungulate densities in the strictly protected part of the BNP are for most species higher than in the managed part. According to the last estimate based on drive counts in 2010 (T. Borowik, unpubl. data) they were: 12 red deer km^-2^, 10 wild boar km^-2^, 2 roe deer km^-2^ , 0.8 bison km^-2^ and 0.04 moose km^-2^. 

 Natural predators of these ungulates, wolf and lynx, are strictly protected in BPF since 1989 and are not hunted throughout Poland. In BPF, they occur with average densities around 2-3 individuals per 100 km^2^ (wolf) and 1-3 individuals per 100 km^2^ (lynx) [49]. The diet of wolf in the area is dominated by red deer (72% of diet) with wild boar as a secondary species (22 %) [39]. Lynx is specialized in roe deer (62 % in their diet) with red deer as the second most important prey (22 %). Wild boar is rarely eaten by lynx (4 % of the diet, [40]).

### Study 1: Perceived predation risk and habitat visibility

To test whether habitat visibility influences ungulate behavior, we collected behavioral data of ungulates by means of camera traps placed on 29 random plots (7 × 7 m and marked by four wooden poles) located in the strictly protected parts of the BNP. As no hunting and forestry activities are allowed in this area and human access is limited, disturbing factors on ungulate behavior are minimal. These plots serve as control plots for a long-term study on the impact of ungulates on tree regeneration and each has a paired exclosure plot consisting of a 2-m high mesh-wire fence surrounding the 7 × 7 m sample plots (see 50 for a detailed description of the experimental set up). Control plots were situated on average 20 m away from their paired exclosure plot with a minimum distance of 5 m. The location of each control-exclosure pair was chosen randomly by placing a grid of 460 cells of 100 × 1000 m over the map of the strictly protected zone, with the shorter side of each cell aligned according to 330° azimuth. Each intersection of the raster was given a unique number and exclosure locations were determined by randomly selecting intersection numbers (see 51). As these plots are randomly located in the area, they cover the natural variation in forest types and abiotic conditions and give a representative sample of the area. To minimize the effect of confounding factors we restricted our analyses to those plots which were located in deciduous and mixed deciduous forest (dominating tree canopy species: *Carpinus betulus, Tilia cordata, Quercus robur, Acer platanoides*) and excluded coniferous, mixed coniferous and streamside alder-ash forest. Hence, we studied the effect of habitat visibility in (mixed) deciduous forest which is the main habitat for red deer and wild boar in this area [47]. This resulted in 24 plots (hence *N* = 24 for study 1) on which we placed camera traps in the period March 2008 - November 2010 to estimate ungulate visitation and behavior at these fixed locations. We excluded recordings from winter (December-February) when trees and shrubs are without leaves, since the visibility measurements (see below) were done in June-July and values likely differ largely between the seasons with and without leaves on trees. We used DVREye™ equipped with Sony CCD video cameras, triggered by both movement and body heat. During low light conditions the cameras switched automatically to infrared illumination which allowed behavioral analyses both at day and night. Preliminary tests showed that the sensors had a detection range of 24–27 m in an area of circa 10°. We placed the cameras at a height of c. 100 cm attached to a tree and at an angle such that the total plot was visible on the recordings. We continuously rotated a set of 12 camera traps among these plots and left them on average for 14 days on each plot. This resulted in 94 red deer visits and 72 visits of wild boar during on average 69.3 recording days (± 3.0 SE) per plot. For each individual red deer and wild boar on the recordings, behavior was determined as described below (see '*Behavioral analyses*') with the exception that only four behavior classes were scored; foraging, vigilance, moving (including both walking, running and sudden rush) and other. Besides, we calculated visitation rate and cumulative visitation time per species per plot (see under 'Behavioral analyses').

We measured habitat visibility at each plot between June-July 2009 (leaves fully developed) as the reverse of the horizontal foliage density [52]. We erected eight transects running from the centre of each plot along the cardinal and subcardinal directions of a compass. We always included in one of these transects the exclosure as a potential escape impediment and/or object blocking the view (see 18). For this we sometimes had to adjust the direction of all transects to keep them equally-spaced. We placed a white pole (200 cm high and 10 cm wide; marked in eight rectangles of 25×10 cm) in the middle of the plot and recorded for each height class the distance at which half of the rectangle was obscured by vegetation or other objects along each of the transect lines. This resulted in 64 measurements of habitat visibility per plot. We calculated the average habitat visibility for the height class 0-175 cm per plot. We used these height classes as they approximate the range in which adult red deer (the larger of the two ungulate species in this study) can scan their environment for potential predators, while foraging or being vigilant (head up). This measure of habitat visibility includes also objects that are regarded as 'escape impediments' and potentially hinder escaping ungulates, such as downed logs, coarse woody debris, dense shrubs or standing trees [24,25]. 

### Study 2: Perceived predation risk and olfactory cues of predator

In the second study we tested whether ungulates react to a cue indicating recent predator presence by comparing ungulate behavior on plots with and without a single added wolf scat. We randomly selected plots inside forest gaps (not connected to the plots mentioned under study 1), that were used only once, throughout the entire managed part of the BPF. We used forest gaps as they have higher ungulate visitation [53]. These forest gaps were anthropogenic clear-cut areas ranging from 1000 to 4000 m^2^ in size. In each forest gap we positioned a camera trap aimed at the centre of the forest gap and attached to a tree at 70-100 cm height. We randomly assigned it as being a control plot or experimental plot (with wolf scat). We used a pair-wise design in which we erected pairs of control-experimental plots in similar forest types and in vicinity of each other (minimal distance between pairs 50 m, maximum distance c. 500 m). As we expected a local effect of the scat, we used a distance of minimally 50 m between experimental and control plots to minimize potential effects of predator odor at control plots. On the experimental plot we placed once one fresh wolf scat in the center of the detection area of the camera, at a distance of c. 10 m from the camera. Fresh wolf scats were obtained from six captive wolves that were mainly fed with wild ungulate carcasses. The required amount of scats for each series of plots were collected every 1-2 days in the wolf enclosure ensuring they were fresh (less than two days old). The exact time of deposition of scats was not known.

 For this experiment it was essential to have records of wild free-ranging ungulates which visit the small plots by chance. Therefore, to gain a large enough sample, we had to replicate these experiments many times between 2009 and 2012 in four periods: 14 October - 4 December 2009, 14 April - 7 June 2010, 28 July - 6 August 2010 and 13 October - 20 November 2012. These periods excluded the rutting season of red deer and winter months with snow cover. This resulted in 21 random controls and 24 wolf-scat plots of which video recordings could be used as we lost some plots due to camera failure. The majority of these paired plots were erected in autumn (September-November, controls: *N* = 13, wolf-scat plots: *N* = 16) followed by spring (March-May, *N* = 5 for both controls and wolf scat plots) and a low number in summer (June-August, *N* = 3 for both controls and wolf scat plots), excluding all plots where camera failed.

 We used two types of camera traps for this experiment: DVREye™ equipped (with Sony CCD video camera) from 2009 to 2010 and Ecotone SGN-5220 in 2012, both equipped with infrared illumination and a similar detection range. To exclude effects of camera type, we always had a similar model erected at a wolf scat plots and its paired control plot (resulting in 11 paired wolf scat-control with Ecotone and 10 control 13 wolf scat plots with DVREye™). We ensured an unblocked view within the detection range of each camera inside each forest gap (distance from camera 27 m in an area of 10°). We monitored all paired plots for 7-10 days and analysed behavior of each individual inside the forest gaps that triggered the camera. Additionally, in autumn 2012 we continued to record ungulates on wolf scat plots only (*N* = 11) for up to five weeks after wolf scat addition, to test for duration of ungulate behavioral responses. 

### Behavioral analyses

We classified behavior of prey species using the following behavioral classes based on the classification schemes used by [9] with some additional categories: (1) *vigilance* was classified when the individual was standing still with its head held parallel to body or higher, looking around and/or twitching the ears occasionally without chewing, (2) *foraging* includes grazing (eating grass or forbs), browsing (eating woody species) or rooting (only wild boar), (3) *walking* (while not feeding or chewing), (4) *running*, (5) *sudden rush* when an animal went from standing still to running, (6) *sniffing* was defined as an animal having its head near the ground, not feeding or chewing, while smelling, (7) *other behavior* included all other types of behavior such as scratching or rubbing against trees, and (8) *checking camera*; walking to camera, sniffing it. We classified behavior of each individual ungulate (red deer and wild boar) within the detection range of the camera inside the forest gap. We grouped behavioral analyses from subsequent videos that were clearly from the same individual (by visually assessing species, sex- and age class) when they occurred within a 5-minute interval from each other. An interval longer than 5 minutes between two recordings was considered as two separate visits. As piglets and young calves together with hinds show behavior that is largely determined by the adult mother, we excluded them from the analyses.

 We determined the time (in seconds) we observed a certain behavior per individual ungulate and calculated the percentage (% of time) of each behavior in relation to the total time that behavior was classified (depending on quality of video recordings and visibility of ungulates). Additionally, we determined the total time that each individual ungulate was present (visitation duration) in the forest gap (including unclassified behavior). Many visits lasted only few seconds. To allow for a reasonable estimate of the percentage of time per behavioral category, we removed all recordings of individual visits shorter than 10 seconds. To obtain visitation rate per plot, we summed the total number of observed individuals per species and divided this by the total days of monitoring of that plot. For this we used all visits (including those < 10 sec). Finally, we calculated the cumulative visitation time per plot per species by summing the duration of all individual visits and correcting for the number of days each plot was monitored.

### Statistical analysis

Regarding study 1, we used linear regression to test for the relationship between habitat visibility and red deer and wild boar visitation rate (number of visits per day) and cumulative visitation time (total time species were present corrected for days of recordings) at each location (plot). Our measures of habitat visibility were normally distributed (Shapiro-Wilk test: *W* = 0.9708, *N* = 24, *P* = 0.686), but we square root transformed all response variables to improve normality. We included each location as a replicate (*N* = 24). 

 To test for the effect of habitat visibility on vigilance level and visitation duration of individual red deer and wild boar we used a LM (Linear Model). As not all locations were visited by all ungulate species, and some locations had low visitation, we used each ungulate visit (red deer: *N* = 94, wild boar: *N* = 72) as replicates for these analyses and included all observations in which we were able to classify the behavior for 10 seconds or longer. We argue that observations can be regarded as independent samples because distance between sites was at least 310 m, covering in total an area of 47 km^2^. As home ranges of the studied ungulates largely overlap between individuals [54,55], several different individuals of each species are expected to occur at each plot during the study period. Since vigilance levels and hence perceived risk effects may vary between seasons (spring, summer, autumn) and from day to night, we included these as factors and their potential interactions in our LM. Arcsinus transformed percentages of vigilance level [56] were used as response variable in the LM and habitat visibility as a covariate. For these analyses we used R version 2.15.2 (R Development Core Team 2013). 

 Regarding study 2, we included next to vigilance also other behavioral categories in the analyses as we were interested if a trade-off existed between vigilance level and other types of behavior. In many occasions no ungulates were present during the recording period, which yielded an estimate of ungulate visitation but resulted in a lost replicate for the behavioral analyses. As a result, data resulting from the wolf scat experiment were strongly non-normally distributed (Kolmogorov-Smirnov tests: *P* < 0.05) and had a very unbalanced design. Therefore, we could not use parametric tests without strongly violating its assumptions and could not combine all analyses in one test showing interactions between factors. Alternatively, we used the following steps to exclude possible confounding factors.

As we expected the strongest behavioral response in the first days after the wolf scat had been deposited on the experimental plots, we firstly analysed only those recordings of red deer and wild boar from the first week of the experiment. We calculated the average visitation rate for red deer and wild boar (number of visits per time interval) and cumulative visitation time (total time red deer or wild boar were present inside the forest gaps) and used plot as replicate and tested for treatment effects using Mann-Whitney U tests since data were non-normally distributed (One-Sample Kolmogorov-Smirnow test, P < 0.05). 

 For the behavioral analyses, we grouped all classes of behavior which comprised less than 1 % (running, sudden rush, checking camera) into the group 'other'. We first tested whether there were seasonal differences in behavior or effects of day and night (one hour after sunset, one hour before sunrise). In case of red deer we combined all data from all seasons (spring *N* = 7, summer *N* = 5, autumn *N* = 71) in which the experiment was carried out since the average of each behavioral category that we determined did not differ between seasons (Kruskal-Wallis for all behavioral categories *P* > 0.225, df = 2). As there were also no differences in any of the types of behavior between day and night recordings (Kruskal-Wallis, for all behavioral categories *P* > 0.325 df = 2) we combined all data to test for treatment effects. In case of wild boar, behavior of individuals recorded in summer (*N* = 6) differed significantly from those recorded in spring (*N* = 20) and autumn (*N* = 105). Compared to the other seasons, wild boar in summer, spent more time foraging (spring: 50% ± 8 SE, summer: 85% ± 6 SE, autumn 41% ± 4 SE, χ^2^ = 8.831, df = 2, *P* = 0.012) and were longer present inside the forest gaps (spring: 35.1 s ± 5.6 SE, summer: 91.0 s ± 13.0 SE, autumn 32.4 s ± 2.0 SE, χ^2^ = 13.950, df = 2, *P* = 0.01). Since all summer recordings occurred on wolf scat plots, we removed them from subsequent analyses. Behavioral categories in spring did not differ from those in autumn (*P* > 0.05). There were no differences in wild boar behavior between day and night, so we combined all data. For both red deer and wild boar sample sizes were too small to allow for testing the interactive effects of treatments and either season or day and night.

 To test for treatment effects on behavior, we abandoned our pair-wise design of the experimental plots, since lack of recordings from either one plot of each pair (due to camera failure or lack of visiting ungulates) resulted in a unbalanced data set. Instead, we treated each individual visit of an ungulate as a replica in the statistical analyses. We acknowledge that male and female red deer may differ in vigilance level in response to wolf presence [9], but due to small sample sizes we grouped different sexes together. We used non-parametric test (Mann-Whitney U test) for treatment effects as most behavioral parameters were non-normally distributed (One-Sample Kolmogorov-Smirnov test, *P* < 0.05). Finally, we tested for how long effects of wolf scats were visible by comparing average behavior of red deer and wild boar that were recorded during the first week and second week on controls and wolf-scat plots. We tested only those behavioral classes that were significantly affected during the first week (vigilance, foraging, sniffing of scat) by means of Mann-Whitney U tests. As an indication of the duration of effects of wolf scat on behavior we analysed whether differences in these behavioral categories occurred during the five weeks that a subset of wolf scat plots (*N* = 11) was monitored by means of Kruskal-Wallis tests. This could only be done for the wolf scat plots, since control plots were monitored up to two weeks only.

 All percentage data (percentage of total time different types of behavior were shown) were arcsine transformed prior to analyses [51], untransformed data are shown in graphs. All analyses of study 2 were performed using SPSS statistical package, version 17.0. 

## Results

### Study 1: Perceived predation risk and habitat visibility

A relatively small range in habitat visibility was recorded within the study area; from an average of 5.3 m ± 0.8 (± SE) to 19.9 m ± 1.1. These differences reflect the heterogeneity that can be found within deciduous forest types in the area and are mainly related to whether a canopy gap is present, tree density, the abundance of undergrowth and the presence of escape impediments (uprooted trees, logs etc.). 

 Neither visitation rate (number of visits per day) nor cumulative visitation time (corrected for days of recordings) of red deer was related to habitat visibility at the location (*F*_1,22_ = 0.932, *P* = 0.345; *F*_1,22_ = 0.040, *P* = 0.843, *N* = 24 respectively). The visitation duration of individual red deer and the percentage of time deer spent vigilant were not affected by habitat visibility at the locations, nor did the effect of habitat visibility interact with season or day/night (see Table 1). Although not significant (*P* = 0.084), visitation duration tended to decrease with increasing habitat visibility (coefficient of regresssion: -1.82, intercept: 67.04). The significant effect of season on vigilance level is caused by the lower vigilance in spring (16% ± 3.4 SE, *N* = 60),compared to summer (31% ± 7.3 SE, *N* = 21) and autumn (32% ± 9.5 SE, *N* = 13) likely explained by higher percentage time spend foraging when food availability is lower at the beginning of the growing season. 

**Table 1:** Behavior of red deer in relation to habitat visibility (study 1).

**Behavior red deer**	**Df**	***Sum of Squares***	***Mean Sum of Squares***	***F***	***P***
**Visitation duration**					
Visibility	1	5674	5674	3.050	0.084
Season	2	322	161	0.087	0.917
Day/night	1	4805	4805.5	2.583	0.112
Visibility × Season	2	2205	1102.3	0.592	0.555
Visibility × Day/night	1	2668	2668.2	1.434	0.234
Season × day/night	2	439	219.3	0.118	0.889
Residuals	84	156295	1860.7		
**Vigilance (%)**					
Visibility	1	909	908.8	1.450	0.232
Season	2	3984	1991.9	3.179	**0.047**
Day/night	1	8	8.2	0.013	0.909
Visibility × Season	2	300	149.9	0.239	0.788
Visibility × Day/night	1	286	286.3	0.457	0.501
Season × day/night	2	104	52.1	0.083	0.920
Residuals	84	52640	626.7		

Statistical results of the Linear Model (main effects of full models) to test for the effects of habitat visibility (between 0-175 cm, entered as covariate), season (categorical explaining variable, three seasons) and day and night (categorical explaining variable) on behavior (visitation duration and % vigilance) of individual red deer (n = 94). Note that the three-way interaction term was removed in this model as it could not be correctly calculated because of the lack of data in some categories. Behavior was expressed as the percentage of time individuals spent on each behavioral category (foraging, vigilance, walking, other) relative to the total time behavior was determined during one visit. Percentage data were arcsine transformed before analyses.

 Similar to red deer, visitation rate and cumulative visitation time of wild boar was not related to habitat visibility at the locations (*F*_1,22_ = 0.462, *P* = 0.504; *F*_1,22_ = 0.010, *P* = 0.922, *N* = 24 respectively). Habitat visibility only directly affected visitation duration, with shorter visits when visibility at locations increased (coefficient of regression: -9.67, intercept: 133.2). Wild boar visitation duration (in seconds) was also longer (significant effect of season in Table 2) in spring (37.4 ± 7.0, *N* = 22) than in summer (31.3 ± 3.5, *N* = 12) and autumn (32.1 ± 5.0, *N* = 38) and concentrated more in low-visibility habitats in autumn compared to the other seasons (see significant interaction season × visibility in Table 2). Vigilance level of wild boar was neither affected by habitat visibility nor by the interaction with season or day/night.

**Table 2:** Behavior of wild boar in relation to habitat visibility (study 1).

**Behavior wild boar**	**Df**	***Sum of Squares***	***Mean Sum of Squares***	***F***	***P***
**Visitation duration**					
Visibility	1	5597	5597	7.661	**0.007**
Season	2	473	237	0.324	0.725
Day/night	1	2669	2669	3.653	0.061
Visibility × Season	2	4989	2494	3.414	**0.039**
Visibility × Day/night	1	249	249	0.341	0.561
Visibility × Season × Day/night	2	2319	1159	1.587	0.213
Season × day/night	2	1659	830	1.135	0.328
Residuals	60	43837	731		
**Vigilance (%)**					
Visibility	1	76	75.7	0.674	0.415
Season	2	314	156.8	1.397	0.255
Day/night	1	2	1.5	0.013	0.908
Visibility × Season	2	92	46.2	0.412	0.664
Visibility × Day/night	1	70	69.6	0.620	0.434
Visibility × Season × Day/night	2	21	10.6	0.094	0.910
Season × day/night	2	3	1.3	0.012	0.100
Residuals	60	6730	112.2		

Statistical results of the Linear Model (main effects of full models) to test for the effects of habitat visibility (between 0-175 cm, entered as covariate), season (categorical explaining variable, three seasons) and day and night (categorical explaining variable) on behavior (visitation duration and % vigilance) of individual wild boar (n = 72). Behavior was expressed as the percentage of time individuals spent on each behavioral category (foraging, vigilance, walking, other) relative to the total time behavior was determined during one visit. Percentage data were arcsine transformed before analyses.

### Study 2: Perceived predation risk and olfactory cues of predator

Although the average visitation rate during the first week of the experiment by red deer was on average 2-fold higher on the control plots (0.43 red deer day^-1^ ± 0.24 SE) compared to the wolf-scat plots (0.21 red deer day^-1^ ± 0.11), this difference was not statistically significant (*Z* = -1.133, n_1_ = 22, n_2_ = 24, *P* = 0.257). Similarly, cumulative visitation time of red deer during the first week did not statistically differ (*Z* = -0.542, n_1_ = 22, n_2_ = 24, *P* = 0.588) between controls (72.8 seconds ± SE 32.7) and wolf-scat plots (33.0 seconds ± 14.5). Also wild boar visitation rate (control: 0.57 wild boar day^-1^ ± 0.17, wolf scat: 0.51 wild boar day^-1^ ± 0.14) and cumulative visitation time (control: 94.2 sec. ± 33.1, wolf scat: 106.3 sec. ± 33.1) during the first week was not significantly reduced on the wolf scat plots (*Z* = -0.266, n_1_ = 22, n_2_ = 24, *P* = 0.790; *Z* = -0.578, n_1_ = 22, n_2_ = 24, *P* = 0.563, respectively).

**Figure 1:**
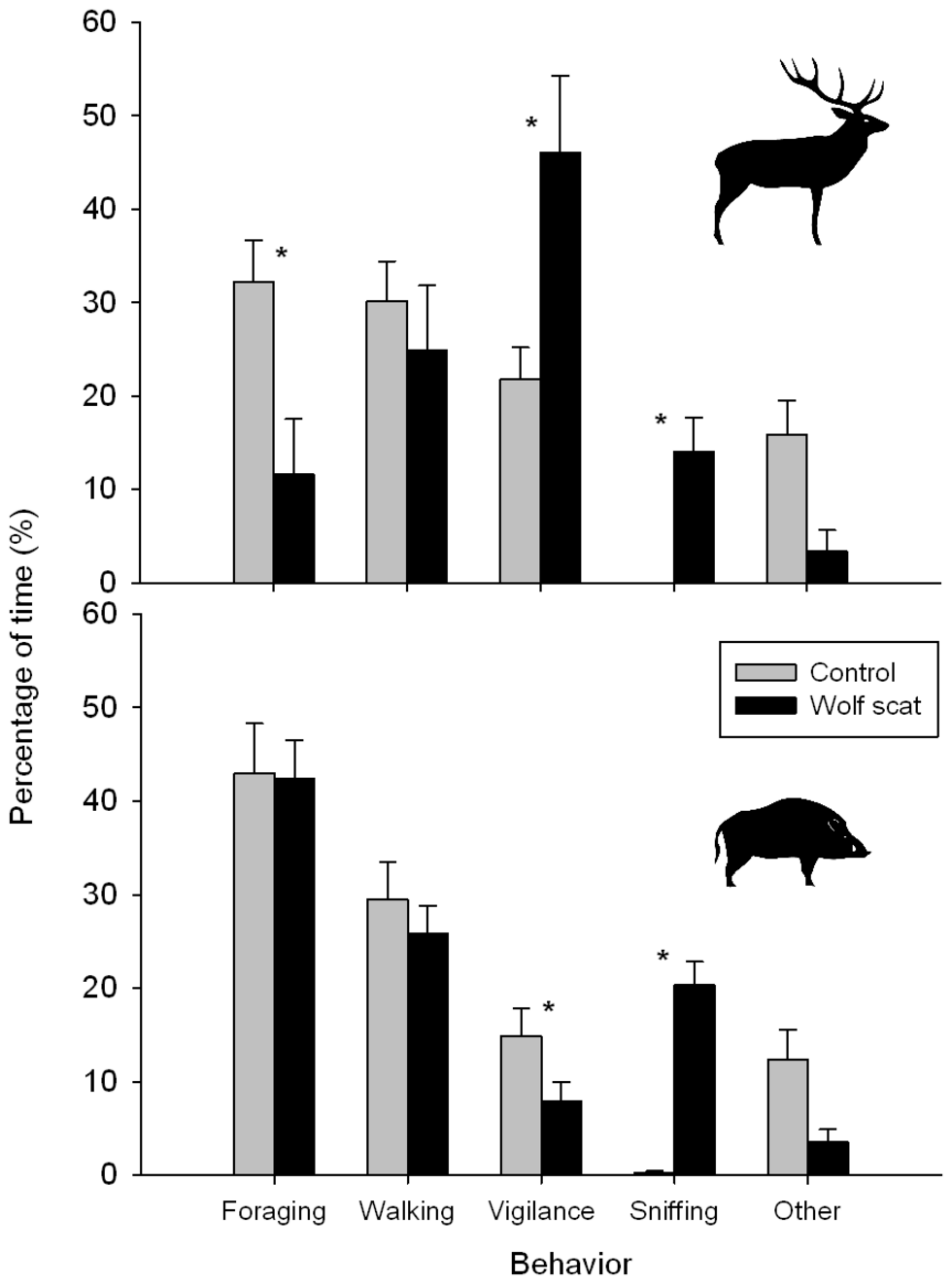
Behavioral response of red deer and wild boar to predator scent. Average percentage of time red deer (upper panel) and wild boar (lower panel) spent on different behavior types (± SE) on control plots (grey bars) and plots with fresh wolf scat (black bars). Numbers are based on 67 and 16 visits of red deer and 55 and 70 visits of wild boar in control and wolf scat plots respectively. Significant differences between control and wolf scat plots within each behavioral category are indicated by the asterisks (at *P* < 0.05 with Mann Whitney U-test).

 The duration of visits of individual red deer did not significantly decrease on wolf scat plots (*Z* = -0.820, n_1_ = 67, n_2_ = 16, *P* < 0.412). However, red deer showed a clear behavioral response when a fresh wolf scat was present in their direct vicinity (Figure 1). They showed a more than 2-fold increase in the percentage of time being vigilant on a wolf scat plot from 22 % on control plots to 46 % on wolf scat plots (*Z* = -2.680, n_1_ = 67, n_2_ = 16, *P* = 0.007). The increased vigilance came at the cost of a similar reduction in time of foraging (*Z* = -2.680, n_1_ = 67, n_2_ = 16, *P* = 0.007) which decreased from 32 % on controls to 12 % when a wolf scat was present. Red deer also spent a considerable higher percentage of time (14 %) on sniffing the location of the scat (*Z* = -6.840, n_1_ = 67, n_2_ = 16, *P* < 0.001). Other behavioral categories did not differ between controls and wolf scat plots (*Z* = -1.460, n_1_ = 67, n_2_ = 16, *P* = 0.144, Figure 1).

 In contrast to red deer, foraging by wild boar was not reduced when a wolf scat was present, whereas the percentage of vigilance even decreased from 15 % on control plots to 8 % on wolf scat plots (*Z* = -6.840, n_1_ = 55, n_2_ = 70, *P* < 0.001, Figure 1). This lack of response in the presence of predator smell is not related to the lack of awareness, because wild boar were sniffing the wolf scat 20 % of the time as compared to less than 1 % spent on sniffing on control plots (*Z* = -6.788, n_1_ = 55, n_2_ = 70, *P* < 0.001). Other types of behavior and also the visitation rate and time did not differ between controls and wolf scat plots (Mann-Whitney U tests*, P* > 0.05).

**Figure 2:**
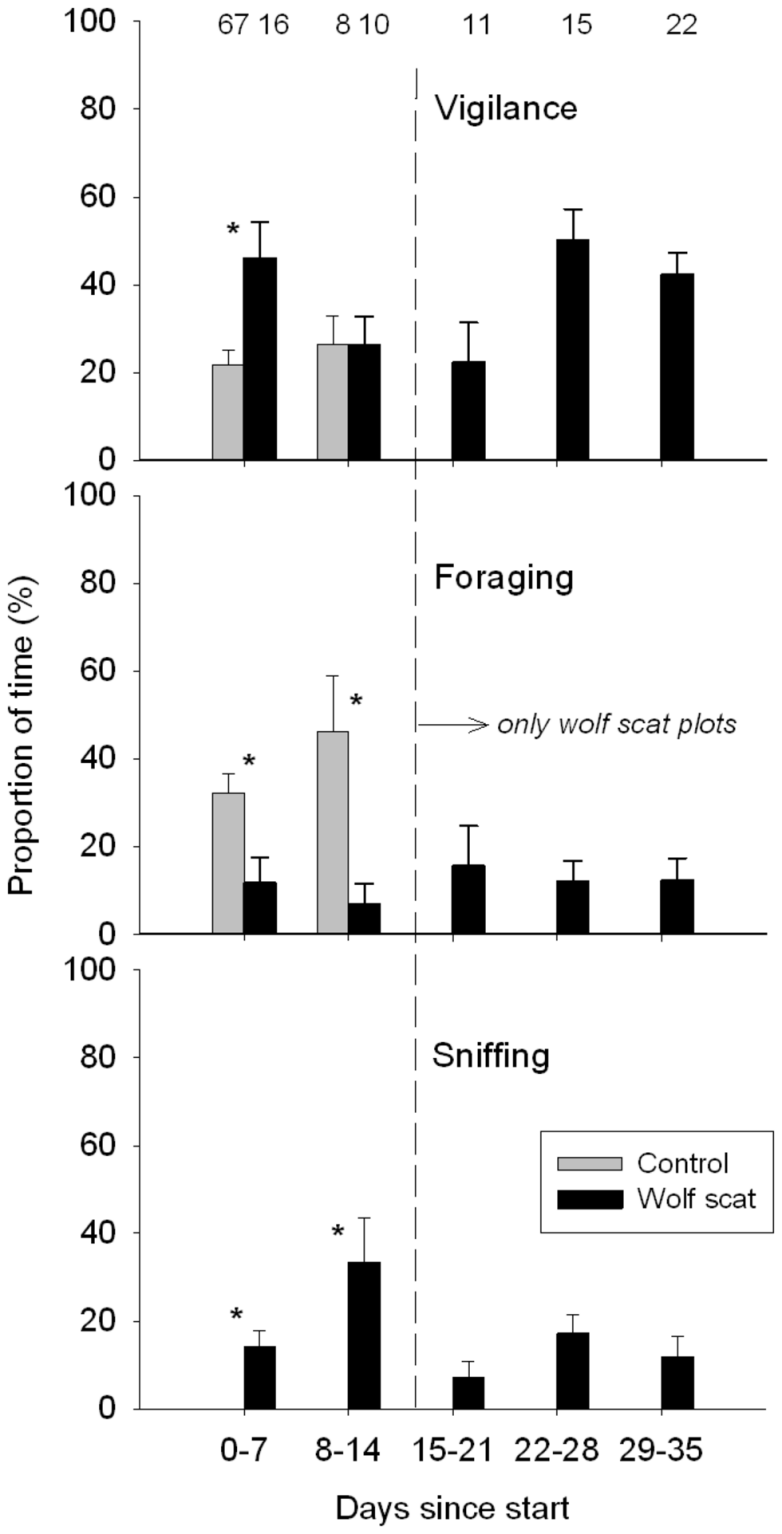
Duration of behavioral response of red deer to predator scent. Average percentage of time (± SE) red deer were vigilant, foraging and sniffing on control plots (grey bars) and plots with fresh wolf scat (black bars). Behavior is shown for the first and second week of the experiment, when both control and wolf scat plots were present. Subsequently, only wolf scat plots have been monitored up to 5 weeks after adding wolf scat. Significant differences between control and wolf scat plots within the first and second week are indicated by the asterisks (at *P* < 0.05 with Mann Whitney U-test).

 Vigilance of red deer on the wolf scat plots was reduced during the second week (8-14 days after scat deposition) of the experiment to a similar level as was observed on the control plots (*Z* = -0.356, n_1_ = 8, n_2_ = 10, *P* = 0.722, Figure 2). However, the percentage of time spent foraging was still significantly lower (*Z* = -2.536, n_1_ = 8, n_2_ = 10, *P* = 0.011) on wolf scat plots (7%) than on control plots (46%) during the second week after addition of the scat (Figure 2). Moreover, the percentage of sniffing was higher on scat plots (*Z* = -2.540, n_1_ = 8, n_2_ = 10, *P* = 0.011) indicating that the scent continued to elicit a response. During the subsequent weeks up to the fifth week, when only the wolf scat plots were monitored, the percentage of time spent foraging and sniffing by red deer did not show a change (Figure 2, foraging: χ^2^ = 1.173, df = 4, *P* = 0.882; sniffing: χ^2^ = 7.133, df = 4, *P* = 0.129).

## Discussion

Although behavioral responses of ungulates to predation risk by carnivores have been extensively studied, the cues that ungulates use to assess predation risk and the scale at which they operate are still under discussion [16-18,57]. We found no indication that habitat visibility is related to perceived predation risk of two common ungulates in densely forested ecosystem. In contrast, vigilance levels of red deer increased at the cost of foraging when confronted with the scent of their main predator (wolf), whereas wild boar (secondary prey species) did not show any response indicating higher perceived predation risk. The response of red deer is particularly remarkable as it was elicited with a single wolf scat. As our study area, the BPF, largely contrasts with previously studied ungulate-predator systems, we provide a first exploration of which factors are important in determining perception of predation risk by ungulates in a densely forested ecosystem. 

### Perceived predation risk and habitat visibility

Earlier studies on ungulate-prey interactions in North-American [21,23-25], African [22,26,32] and European [31] ecosystems showed clear relationships between habitat visibility and predation risk effects. In these studies, ungulates showed behavioral responses which indicate increased perceived predation risk, such as avoidance of high risk areas (with high proportion of kills), increased group size or a higher percentage of time spent on vigilance. In case of red deer in our study, neither were visitation rate and cumulative visitation time at a location nor visitation duration and vigilance level of individual red deer related to habitat visibility. The lack of a relation between habitat visibility and behavior indicate that red deer in dense forests do not perceive habitats with a lower visibility as more risky. In case of wild boar, habitat visibility only directly affected visitation duration with longer visits at locations with lower visibility. This is likely explained by preferential foraging under tree canopy (in contrast to tree canopy gaps with higher visibility) where seed rain by trees, especially acorns, is higher [47]. This is confirmed by the longer visits and the higher concentration in the more low-visibility habitats in autumn compared to the other seasons (significant interaction season × visibility). As in autumn acorns (and other tree seeds) form a principal food source for wild boar, a higher concentration in low visibility habitats is expected. Since no relationship existed between habitat visibility and time spent vigilant by individual wild boar, we do not interpret this as indicative for predation risk effects. As both red deer and wild boar showed similar vigilance levels in similar habitats (all deciduous forest types) which largely contrasted in habitat visibility, it indicates that both species do not perceive forest with low habitat visibility as more risky. 

 These results seemingly contrast to our recent findings that coarse woody debris (CWD, uprooted trees, fallen logs) creates fine-scale risk effects on ungulates in this forest system [58]. These findings suggested that deer reduce foraging in close vicinity to large amounts of CWD by either behavioral changes (higher vigilance at the cost of foraging) or avoidance of these places [58]. Although the measurements of habitat visibility in the present study include these fine-scale risk effects, it mainly expresses the general visibility of the environment at a given location encompassing a larger scale. Habitat visibility as such is mainly determined by the density of trees and undergrowth at a certain location and to a lesser extent by the presence of CWD. Moreover, study plots for which habitat visibility had been determined in the present study were located throughout the Białowieża National Park and thus included locations both inside and outside the core area of the wolf territory present (see 49). In line with the findings of [58], habitat visibility might have a different effect on ungulate behavior inside versus outside a core area of a wolf territory. Although we cannot rule out this context-dependence due to the small sample size of plots, our findings show that habitat visibility is not an important general cue for ungulates to assess predation risk in these densely forested areas.

 A likely explanation for the lack of effect of habitat visibility is that in a dense forest the differences between habitat types are not sufficient to provide ungulates with a reliable indicator of differences in predation risk. Studies that showed a relation between ungulate vigilance and habitat visibility were carried out in open or half-open landscapes [21-26,32]. In contrast, our study area is densely forested with only 0.8% of the area permanently deprived of tree cover and consisting of open grassland [48]. Hence the area in general is characterised by low habitat visibility with relatively small differences between forest types. Moreover, due to the lower landscape heterogeneity and the much smaller area of our study system (c. 600 km^2^, Polish part of BPF), compared with for example the YNP (8.980 km^2^), ungulates cannot move to predator free areas or habitats with lower predation risk. The home ranges of the present wolf packs and lynx territories overlap the entire forest area and predator-free parts of the forest do not occur [49]. This lack of choice for ungulates in our system to move to safe areas or habitats, in contrast to YNP, may result in ungulates using different cues than general habitat visibility to assess predation risk and rather use fine-scale risk factors within habitats as a more important cue [58].

 Finally, the lack of relation between habitat visibility and perceived predation risk by red deer and wild boar might be related to the presence of two types of predators using a different hunting strategy. Wolf is a typical chase-hunter and lynx an ambush-hunter. Experimental studies on invertebrate predator-prey systems showed that predators that use an active (chase) hunting strategy are less likely to exert risk effects related to habitat features than predators that use a sit-and-wait (ambush) hunting strategy [59]. Similar differential effects of predator hunting mode in large carnivore-ungulate systems have been suggested to occur [17,19] and recent African studies found evidence for this [32]. Increasing vigilance levels of ungulates with decreasing habitat visibility have been demonstrated directly in areas where ambush-predators such as large felids were present [22,26,32], whereas direct evidence for this is less common from wolf-dominated systems [21] and may likely have the opposite pattern [18]. In our study area deer constitute an important prey species for both wolf and lynx, whereas wild boar is only consumed by wolf [39,40]. As lynx requires good cover while stalking the prey [29], it may lead to higher vigilance levels of their prey in low-visibility habitats, whereas no or the opposite effect might occur for chasing predators as wolves that make most kills in high-visibility habitats [10,20]. As red deer is under predation pressure of both predators, the habitat-linked risk effects exerted by lynx could be opposed by wolves, leading to no measurable relationship between habitat visibility and deer vigilance levels. 

 To conclude, we found no support for the hypothesis that ungulates use habitat visibility as a cue for predation risk in dense forest ecosystems. Whereas fine-scale habitat-linked risk effects do occur in our area (see 58), visibility in general does not seem to be used by ungulates to assess predation risk in these closed habitats.

### Perceived predation risk and olfactory cues of predator

Olfactory cues likely play an important role in dense forest systems and we predicted that ungulates should either reduce visitation rate or increase vigilance levels after being exposed to cues of recent presence of a predator. In accordance with this, red deer, the main prey species of wolf in the BPF [39], clearly responded by largely increasing their vigilance levels in plots with added fresh wolf scat. Red deer showed a more than 2-fold increase in the percentage of time being vigilant when a wolf scat was present; from 22% on control plots to 46% on wolf scat plots. This increased vigilance came at the cost of a similar reduction in time of foraging (from 32% on controls to 12% on wolf-scat plots). While foraging (32% of the time) was the dominant behavior on control plots, this changed towards vigilance (46%) as the dominant behavior when a wolf scat was present. Interestingly, the addition of wolf scat did not result in a lower visitation rate compared to control plots. Typically, deer that visited our experimental plots, sniffed the wolf scat followed by showing alert behavior and scanning the environment (twitching ears, looking around). This response suggests an increased perceived predation risk, operating only at a fine-scale (at a distance of circa 2 m from a scat).

 In contrast to red deer, wild boar did not react to the added wolf scat. Wild boar vigilance levels did not increase and their visitation rate was not altered. The high percentage of time wild boar were sniffing the wolf scat indicates that they were aware of its presence. We explain this lack of fear response by the fact that wild boar is of secondary importance in the wolves' diet in our study area [39] and are a negligible prey of lynx [40]. Moreover, predators remove a smaller proportion of individuals from the population of wild boar compared to red deer [41], and predation weigh less in total mortality of wild boar compared to red deer [42]. As a result of this lower predation pressure they might not perceive cues indicating wolf presence as risky (see also 31). In fact, wild boar seemed attracted by the presence of wolf scat and in nearly all cases sniffed and examined it, while not eating it. Several times wild boar were observed to press their belly into the location where the scat was deposited. Although the reason for this behavior is unclear, it clearly shows that wild boar do not perceive any elevated risk.

 We demonstrated that the effect of a fresh wolf scat on the perceived predation risk of red deer lasts for at least one week but found indications that it may last much longer. In the second week of the experiment, there was no difference in vigilance behavior on control plots versus wolf scat plots, whereas foraging was still significantly reduced. Continuous monitoring of the scat plots only, showed that deer continued to sniff the scat up to five weeks after deposition and foraging behavior did not increase, which indicates that it elicits a response much longer than one week. However, the lack of elevated vigilance level suggests that this behavioral response does not indicate higher perceived predation risk. Deer might only react to a freshly deposited wolf scat as a cue indicating vicinity or recent presence of a predator, hence recognizing a near-imminent threat. This is in line with studies from North American ecosystems [8,19,60] showing that elk changed behavior and movement patterns when wolves were in the direct vicinity within a radius of 3 km, or when wolves had been present at a location less than eight hours ago. As both elk and wolf can be easily observed in some North American ecosystems, direct sighting of the predator by their prey likely play a role in these half-open landscapes [8]. In dense forested areas, where visibility is consequently lower, using olfactory cues might be a more important mechanism for ungulates to assess predation risk. Additionally, our study showed that ungulates can react to fine-scale cues indicating differences in perceived predation risk, for which unambiguous evidence from North American ecosystems is currently still missing [18].

### Implications for the role of carnivores in dense forest ecosystems

Although behavioral changes as a reaction to predator odor have been shown before in wild rodents [61], domestic ungulates [35,36], and marsupials [62], our study shows effects of wolf olfactory cues on a wild ungulate (red deer) in a natural environment and with an ecologically sensible concentration of predator excrements. Considering that ungulates did react to a very low concentrations of the predator's odor, implies it plays a significant role in predator-prey interactions.

 In wolves, the primary function of scent-marking is territory maintenance [63]. Also scat deposition may serve this function [64]. Marking rates per unit of time that wolves spent in a given part of their home range is generally more intense or they are placed at more exposed sites towards the edges of their territory [63-65]. The spatial pattern of scent marking intensity in the Białowieża Forest differed from the ‘olfactory bowl’ model proposed by [63], in which the density of marks was highest and equally distributed along territory edges. Instead, in our study area the highest density of marks (including scats) was observed in the centre (core area) and in some places along the edges of the wolves’ territories [65]. This resulted in marks concentrated in ‘hot spots’ more valuable to owners (such as vicinities of wolf breeding dens) or more vulnerable to penetration by intruders (territory edge). Due to the higher presence of wolves close to their den sites, the density of scat is generally higher inside the wolf core area [64,65]. In this way, predators create spatial patterns in scent marking aimed at intra- or inter-pack communication. The present study suggests that ungulates potentially use this information as well to assess the area where predators are most frequently present.

 The idea that ungulates distribute themselves according to spatial patterns of their main predators and that scent can play an important role in this have been proposed earlier. As wolf packs tend to avoid intensive use of buffer zones, deer inhabiting those areas are predicted to experience lower predation pressure [66] and numbers can become higher at boundaries of wolf packs compared to the territory centers [66,67]. Our study adds to these ideas that also at a more fine-scale, patterns in scent marking within a wolf territory can induce behavioral changes in ungulate prey species. This illustrates the importance of non-lethal effects that a predator can induce in contrast to lethal effects that affect the number of ungulates. As vigilance levels increased at the expense of foraging, this opens up the opportunity for predators to shape (at fine-scale) patterns in ungulate browsing and effects on tree recruitment [50,68] via the 'landscape of fear' they create. 

 The relative importance of direct and indirect predator effects and their role in causing trophic cascading effects has been under continuous debate [17,18,57]. There is increasing awareness that the hunting mode of predators is an important factor modifying the landscape of fear that they produce [32,69]. Predators with an active hunting mode, roaming over large areas such as wolves do, are supposed to be least likely to produce consistent risk cues at any spatial location or habitat type [17,32]. The location where kills occur, and their habitat features, might not necessarily well express the perceived predation risk by ungulates. The location of a kill is a result of a complex set of predator-prey interactions, involving encounter, chasing and eventually killing of the prey [57]. As a result, encounter sites and kill sites may be as far as a kilometer apart [20] and may be de-coupled from habitat features where ungulate prey have highest perceived predation risk. In line with this, the present study showed that fear experienced by ungulates was not related to habitat visibility, despite that both a typical chase-predator (wolf) and an ambush-hunter (lynx) were present in our ecosystem. Moreover, our study suggests that in contrast to what is predicted based on its hunting mode, also a typical chase-predator as a wolf may produce consistent risk effects by leaving olfactory cues. Hence, despite that roaming predators may not create a consistent landscape of fear that can be related to habitat features, they may create patterns in scent marking potentially influencing herbivore-plant interactions.
